# Supplementary material for: New Anti-Flavivirus Fusion Loop Human Antibodies with Zika Virus-Neutralizing Potential
Source: Int J Mol Sci. 2022 Jul 15;23(14):7805. doi: 10.3390/ijms23147805 (PMC9321016; doi:10.3390/ijms23147805)
Supplement: Supplementary file 1 [file ijms-23-07805-s001.zip › ijms-1775354-supplementary.pdf]

Supplementary materials

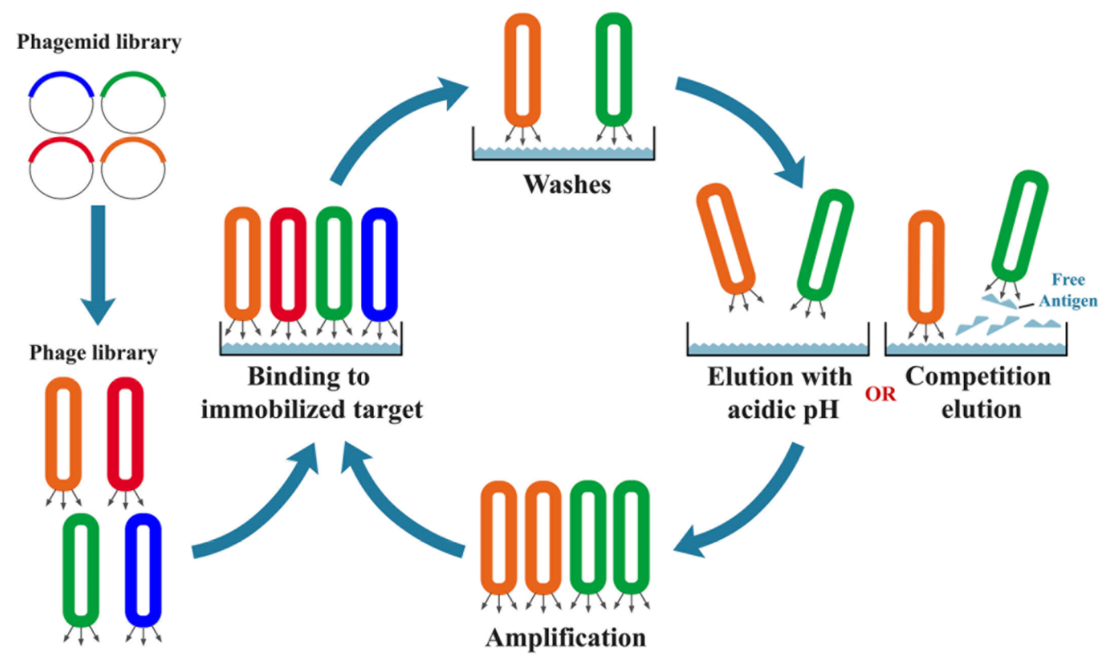

Figure S1. Schematic representation of the selection processes. A combinatorial library was used to generate fusion phages expressing human antibody fragments on their surfaces. Four selection rounds were performed, increasing the stringency at each round by raising the number of washes. Two strategies eluted the specific phages. One used an acidic solution to disfavor antibody-antigen binding. The other used a competitively saturating solution of the free unlabeled FL antigenic peptide. Eluted phages were amplified separately for a new selection round.

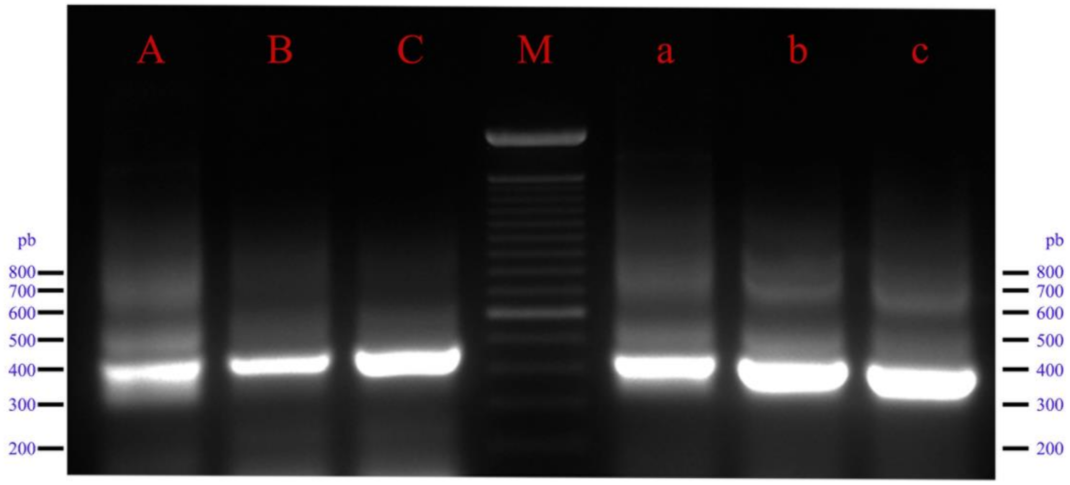

Figure S2. Generation of variable domain genes amplicons. Phagemid pools from the original library and the fourth round of selection were used as templates for PCR using

specific primers for VH (capital letters) and VL (lowercase letters) domains amplification. The amplicons were obtained through antibody library before selection (A, a) and from the fourth round of selection using acid (B, b) or competitive (C, c) elution. M: 100 bp DNA Ladder.

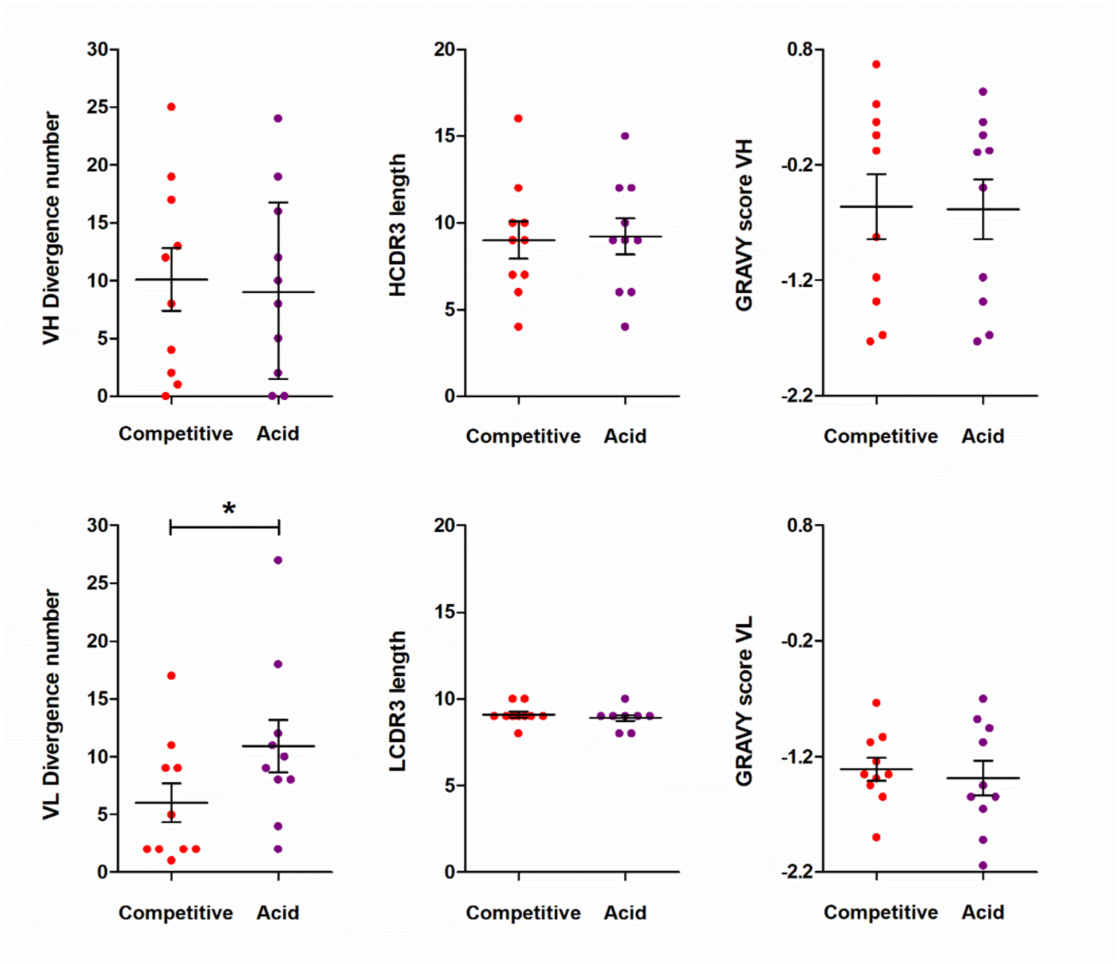

Figure S3. Properties of the most enriched VH and VL. The characteristics of the ten most frequent variable domains in the acid selection (Acid) and competitive selection (CP) were analyzed. The characteristics of VH and VL studied were: divergence to the germline V gene (number of non-identical residues); CDR3 length (number of residues); and GRAVY scores of hydrophobicity of the CDR3 amino acid sequence. Data are represented as the means and SEM in each group (N=10/group). *Means of groups were statistically different at $P < 0.05$; unpaired t-test.

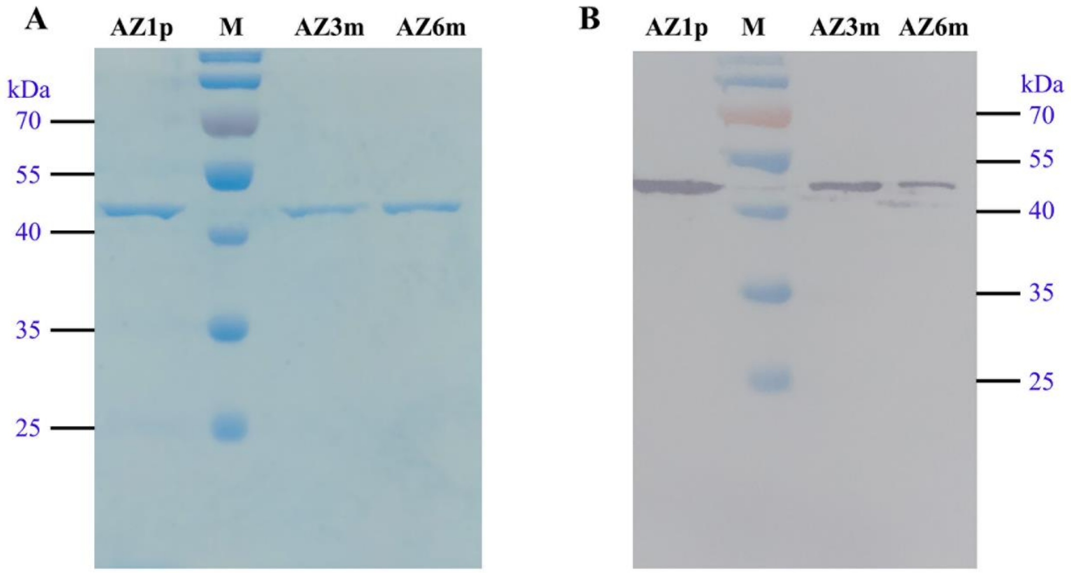

Figure S4. Analysis of IMAC purified recombinant scFvs. The scFv were expressed in bacteria and purified by affinity chromatography in nickel columns. The production of antibodies AZ1p, AZ3m and AZ6m were analysed by denaturing SDS-PAGE (**A**) and detected with anti-HA tag antibody by western-blot (**B**).

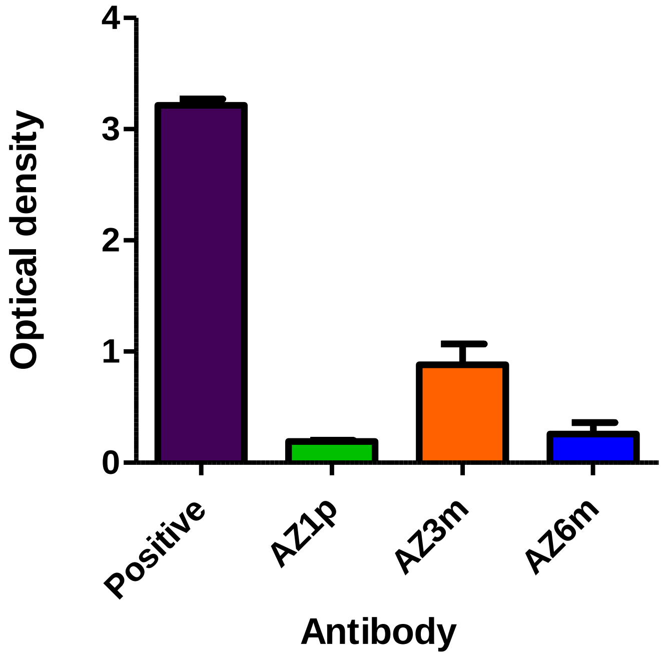

Figure S5. Binding of antibodies to measles virus. The scFvs, AZ1p, AZ3m and AZ6m were analyzed for their ability to bind to inactivated measles virus (viral particle).

Polyclonal measles virus-specific IgG was used as a positive control. The absorbances obtained at the highest concentration tested (approximately 2 μM) of the antibodies are shown. Lower tested concentrations of the scFvs (from 658.5 ηM to 24.4 ηM) resulted in absorbances of less than 0.1.

Table S1. Statistical results of sequencing of the selected variable domains.

Domain population (round)	Total number of sequences	Number of unique sequences	Ratio unique sequences / total number
VH (R0)	64043	55059	85%
VH (R4CP)	72624	18080	25%
VH (R4AC)	15776	5568	35%
VL (R0)	89185	41919	47%
VL(R4CP)	81411	7050	8,7%
VL (R4AC)	92952	10560	11,3%

R0: Round 0 (before selection). R4CP: Round 4 of competitive selection. R4AC: Round 4 of acid selection.
